# The impact of working memory and the “process of process modelling” on model quality: Investigating experienced versus inexperienced modellers

**DOI:** 10.1038/srep25561

**Published:** 2016-05-09

**Authors:** Markus Martini, Jakob Pinggera, Manuel Neurauter, Pierre Sachse, Marco R. Furtner, Barbara Weber

**Affiliations:** 1 Department of Psychology, University of Innsbruck, Innrain 52, 6020 Innsbruck, Austria.; 2 Department of Applied Mathematics and Computer Science, Technical University of Denmark, Anker Engelunds Vej 1, 2800 Kgs. Lyngby, Denmark.

## Abstract

A process model (PM) represents the graphical depiction of a business process, for instance, the entire process from online ordering a book until the parcel is delivered to the customer. Knowledge about relevant factors for creating PMs of high quality is lacking. The present study investigated the role of cognitive processes as well as modelling processes in creating a PM in experienced and inexperienced modellers. Specifically, two working memory (WM) functions (holding and processing of information and relational integration) and three process of process modelling phases (comprehension, modelling, and reconciliation) were related to PM quality. Our results show that the WM function of relational integration was positively related to PM quality in both modelling groups. The ratio of comprehension phases was negatively related to PM quality in inexperienced modellers and the ratio of reconciliation phases was positively related to PM quality in experienced modellers. Our research reveals central cognitive mechanisms in process modelling and has potential practical implications for the development of modelling software and teaching the craft of process modelling.

The processes of ordering shoes from an online store, taking out a loan, booking a flight, or renting a car can be depicted in formalized graphical models called process models (PM; Fig. 1A). PMs are most often applied to organize, optimize, and communicate a company´s business processes. They facilitate organizational processes and support the development of different types of information systems including process-aware information systems, service-oriented architectures, and web services^1^,^2^,^3^. PMs can be found in a variety of different domains including insurance companies, bank institutes, and hospitals.

Various well known factors affect the ease with which a modeller will understand a PM, e.g., modelling expertise and process knowledge^4^,^5^, cognitive abilities, learning style and learning strategy^6^, the modelling notation^7^, as well as model characteristics^8^. While PM comprehension is well understood, only few studies focused on PM creation^9^,^10^. It has been demonstrated that complex PMs tend to contain more errors^11^, and that characteristics of the modelling task influence PM quality^11^. In addition, the importance of modelling knowledge is emphasized^4^,^5^ and first results point towards the impact of the process of creating a PM on its quality^10^. Nonetheless, a more detailed understanding of factors influencing PM quality, and therefore the creation of PMs, is in demand.

The creation of PMs can be characterized as a design activity within the field of problem solving^12^,^13^,^14^. A variety of challenges are imposed, including the construction of a mental model of the domain as well as the externalization of the mental model by mapping it to the modelling elements provided by the modelling notation using a modelling tool. Literature suggests that cognitive abilities such as working memory (WM) are crucial for creating PMs of high quality, for design activities, and problem solving in general^15^,^16^.

The aim of the present study was to obtain a better understanding of the factors leading to PMs of high quality by examining the role of working memory in experienced and inexperienced modellers. As a primary contribution, our research reveals central cognitive mechanisms in process modelling, and has potential practical implications for the development of modelling software and process modelling teaching. In addition, the paper aims to explore the roles of specific phases in the process of process modelling (PPM) while PMs are created. In the following we provide theoretical backgrounds on: (i) process modelling and PPM phases, (ii) PM quality, (iii) WM, and (iv) experience. This is followed by the description of our hypotheses, their statistical testing and discussion. Finally, a breakdown of the methodological approach is presented.

## Theoretical Backgrounds

### Process modelling as a cognitive design activity and PPM phases

**Figure 1:**
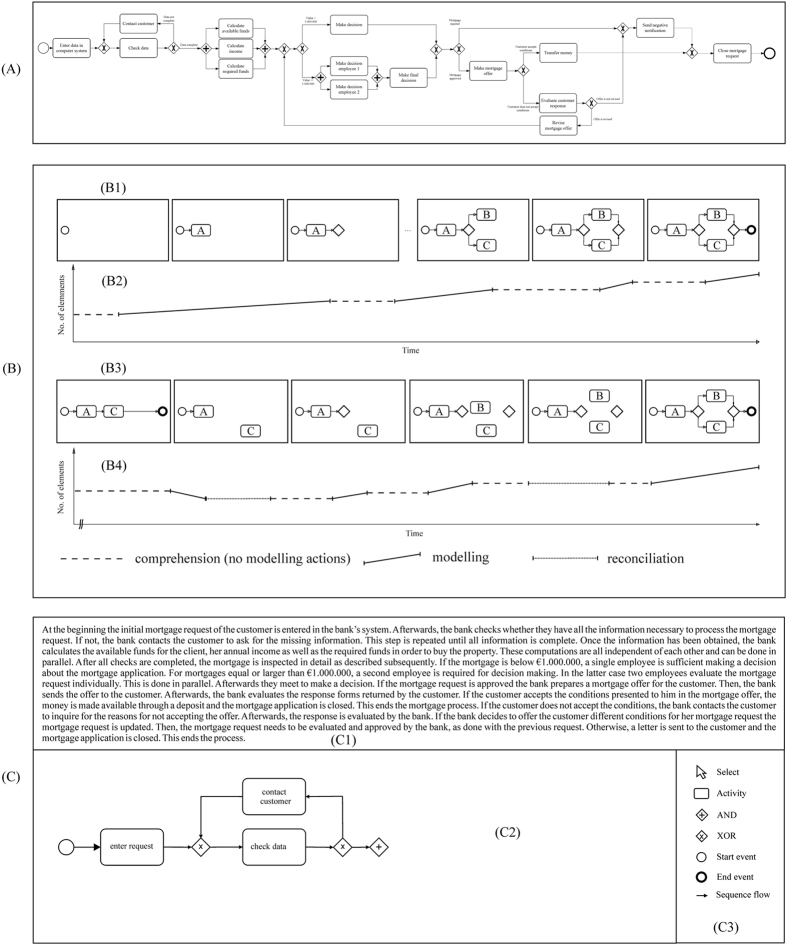
(**A**) Depicted is an example of a (goal state) PM. (**B**) Illustrated are two modelling phase diagrams. (B1,B3) show that based on the same textual process description different creation processes are revealed. In (B2,B4) the x-axis represents time and the y-axis the number of elements in the model. The diagram depicts the number of C-, M-, and R-phases and their length of time. In (B1) a model is created in a straight–forward series of modelling interactions. The model of (B1) starts with adding a start (grey circle) event, followed by adding an activity element A. In turn, in (B3) two activities A and C are created, which are connected to the start and end event in a sequence (black circle). Following this, the modeller realizes that an activity B should be mutually exclusive to activity C. Thus, the modeller has to remove parts of the PM to add the missing model element. Consequently, several model elements are placed in the modelling area. Finally, the PM is laid out to complete the PM (**B** was taken from Pinggera *et al*.^22^) (**C**). Schematic illustration of the CEP modelling environment. The figure shows the textbox for the process description (C1), the modelling area including parts of a PM (C2), and the modelling toolbox with various operators (C3).

Process modelling can be embedded in the PM lifecycle, which involves elicitation and formalization phases^17^. In elicitation phases, information is generated by domain experts, forming the foundation for formalization phases, in which process modellers create a PM^18^. The formalization of a PM can be described as a cognitively demanding problem solving process^13^,^19^,^20^,^21^ where modellers try to transform an initial state into a goal state. For instance, a textual and/or oral description of a process in a specific domain is translated into a formal PM, based on the application of specific operators (e.g. two activities such as “evaluate” and “decide” can be associated with the “AND” gateway; Fig. 1) and their restrictions (only use the “AND” gateway when activities are executed in parallel). The challenges imposed on the modeller have been assigned to three specific phases in the PPM by Pinggera *et al*.^22^. According to Pinggera *et al*.^22^ the PPM can be divided into three phases: comprehension, modelling, and reconciliation. Comprehension (C)-phases imply the understanding of the problem, mapping this understanding to specific modelling constructs of the modelling notation, and validating the (partial) mental and/or externalized model. Modelling (M)-phases define the externalization of the mental model as a PM, i.e. the creation of the PM through interaction with the modelling environment. Reconciliation (R)-phases involve reorganizing activities (e.g. renaming activities) and the usage of secondary notations^23^ (e.g. laying out of activities). The study by Claes *et al*.^10^ points towards the importance of the modelling process for the creation of high-quality models. The role of PPM phases for the creation of high-quality PMs is, however, not yet sufficiently understood and will be investigated as part of this study.

### PM quality

PM quality is used as dependent construct in the present study and assesses the extent to which the final PM represents the requirements of the domain in a syntactically and semantically correct way. The final PM (representing the end state a particular modeller reached) results from a sequence of operator applications. To assess its quality, at least in laboratory settings, this final model can be compared with a model representing the goal state, i.e., the ‘ideal solution’ for this process. To operationalize the difference of the final PM and the goal state model we use two quality measures, graph edit distance (GED) and activity correctness (AC). GED quantifies the transformation steps (i.e. operator applications) required to convert the current PM into the ‘ideal solution’ of the PM^24^,^25^. In turn, AC measures whether activities are identified and represented in a semantically correct way, i.e. whether all activities of the goal state model are included in the final PM as well.

### Working memory

One of the most important cognitive systems for creating a PM is WM. WM defines a system, which retains a limited amount of information in a temporarily accessible state and makes it available for processing^26^,^27^,^28^. For instance, when a PM is created, relevant information of the process to be modelled has to be activated, selected, and maintained and existing knowledge about different PM patterns, solution strategies, and software applications have to be implemented. Interindividual differences in WM capacity limitations have been related to a host of higher-order cognitive processes such as language comprehension^29^, logic learning^30^, fluid intelligence^31^, and the integration of pre-existing domain knowledge^32^ - cognitive abilities of high importance in creating PMs. There is an ongoing debate about the nature of interindividual differences in WM capacity and therefore different explanations exist. For instance, for Engle and colleagues^33^,^34^ WM capacity is best defined as sustained attention in the face of interference or distraction. For Unsworth and Engle^35^ WM capacity refers to active information maintenance in primary memory (i.e. within the focus of attention) and a controlled search and retrieval from secondary memory (i.e. outside the focus of attention, in long-term memory). For Oberauer and colleagues^36^,^37^,^38^ limits arise from interferences between information element bindings (e.g. words or sentences and their relation to each other).

There are a variety of different tasks for measuring WM capacity. In the present study we focussed on measuring two WM functions, (i) concurrent holding and processing and (ii) relational integration. Holding and processing was examined with the operation span (OSPAN) task. This task measures the ability to hold a limited amount of information outside the focus of attention (i.e. letters), while other information is simultaneously processed (i.e. solving arithmetic equations)^39^,^40^. Holding and processing represents a WM function that has most often been related to higher order cognition^29^,^30^,^31^. Relational integration measures the ability to build new relations between elements. This ability was examined with the spatial short-term memory (SSTM) task in which a sequence of dots have to be integrated into a pattern and maintained for a later recall^41^,^42^. Oberauer *et al*.^37^,^43^ found that both WM functions are highly correlated but separable processes and dissociable from a third construct, the so called supervision, which is responsible for attentional focus shifting between task sets^44^.

Within the process modelling literature there is clear awareness of the WM construct, its functions, and possible roles in process modelling^19^,^23^,45–47. However, most often the central importance of WM in process modelling was primarily theoretically implemented. Only a few studies tested the role of WM in PM creation^14^ or related concepts (e.g. learning styles) in understanding PMs^48^. For instance, a direct evidence for a possible role of WM in process modelling was empirically tested by Sachse *et al*.^14^. Taking into account that WM consists of a set of processes, Sachse *et al*.^14^ related three different WM functions to various measures of PM quality. They found that WM updating was negatively related to syntactic errors, and relational integration was negatively related to semantic errors and positively related to GED.

### Experience

A host of studies show that deliberate practice (training) improves performance in various domains such as chess, music, memory, informatics, and sport^49^. Practice can lead to cognitive and behavioural processes of much more accuracy, effectiveness, and ease^50^. One view about the theoretical explanation of this effect is that through practice an increasing number of information units (domain knowledge specific chunks) are generated and heavily interconnected in long-term memory, leading to a much more effective information processing despite limited WM capacity resources^51^. In other words, limited WM capacity resources can be circumvented by an increasing body of domain knowledge that is stored in long-term memory^32^,^49^,^50^,^51^. For instance, in the domain of sport knowledge, Fincher-Kiefer, Post, Greene, and Voss^52^ found that participants high in baseball knowledge outperformed participants low in baseball knowledge on a reading span task (which measures WM capacity for verbal information) when sentences were baseball-related. No differences were found when sentences were domain knowledge free. However, not only the amount of knowledge changes performance. Recent studies show that WM capacity explains additional variance in expert performance. For instance, Hambrick and Engle^32^ found that WM capacity contributed to memory performance even at high levels of knowledge in the game of baseball. In sum, practice together with WM capacity limitations play a relevant role for the development and interindividual differences in expert performance. In the present study we investigated two groups with various amounts of experience in the creation of a PM. Even though empirical evidence exists that modelling experience, education, and knowledge on process modelling are relevant factors for the understandability of PMs^4^,^5^, no study to date investigated the role of cognitive (WM) and PPM factors within and between modellers of various experience levels in the creation (vs. comprehension^4^,^5^) of PMs. More specifically, our study investigated relevant WM factors in addition to relevant PPM phases in the earliest phase of expertise development.

## The Present Study

In line with the theoretical background discussed above, we argue that the quality of PMs depends on the modeller’s process modelling experience, his/her cognitive abilities (i.e., WM), as well as the process that is followed in terms of PPM phases. However, the relations between specific WM functions, PPM phases in creating a PM, and the role of modelling experience is poorly understood. Therefore, we investigated two WM functions (holding and processing with the OSPAN task, and relational integration with the SSTM task^42^) and three PPM phases (C-, M-, and R-phases^22^) in relation to the quality of a PM, in inexperienced and experienced modellers.

Existing studies show that modelling experience is a relevant factor for PM comprehension^4^,^5^. In addition, studies show that experience in a certain domain can improve domain specific task performance^49^. We hypothesized that:

H1: *Experienced modellers create PMs of higher quality compared to inexperienced modellers.*

There are theoretical considerations of WM as a relevant construct for creating PMs^19^,^23^,^45^,^46^,^47^. What remains unaddressed is the question whether there are differences depending on modelling experience. In extension to Sachse *et al*.^14^, the present study investigated the WM functions of concurrent holding and processing of information, and relational integration in relation to PM quality, in experienced and inexperienced modellers. Based on the view that the applied WM tasks are domain knowledge free measures, and existing studies show that WM is related to task performance within inexperienced^14^,^37^ and experienced participants^53^ we hypothesized that:

H2: *Both WM functions are positively related to PM quality in inexperienced and experienced modellers.*

The roles of different PPM phases^19^,^22^ for the creation of high-quality PMs is not yet sufficiently understood, leading to difficulties in formulating strong hypotheses. In the present study, the ratios of the number of C-, M-, and R-phases and total processing times (in the following rC-, rM-, and rR-phases) were related to PM quality for the first time. We assumed that unfamiliar and complex tasks are cognitively demanding and time consuming, especially for inexperienced modellers. Higher rC-phases can therefore be an indicator for problems within the modelling process, for instance, difficulties in text understanding, application of operators, or creating a mental model based on conceptual information. The same can be supposed to be the case for rM-phases. A higher number of rM-phases, i.e. higher numbers of adding and deleting operations can be an indicator for problems during the externalization of the PM. Assuming an ideal modelling process without the modeller facing any problems, a modelling process with just a single rC- and a single rM-phase would be the result. In a first step a goal state model is mentally created within a single rC-phase before the PM is externalized in a second step, within a single rM-phase. Based on this view we hypothesized the following:

H3: *rC-phases are negatively related to PM quality, particularly in inexperienced modellers.*

H4: *rM-phases are negatively related to PM quality, particularly in inexperienced modellers.*

Regarding the rR-phases we assumed that reorganizing and restructuring model elements can be viewed as important steps in creating readable and understandable PMs. This should lead to a higher chance of finding contained errors before the creation of the PM is finished and facilitates the creation-process itself by lowering the demands for the correct positioning and integration of new elements within the PM. Consequently, PMs of higher quality should be created. We hypothesized that:

H5: *rR-phases are positively related to PM quality in inexperienced and experienced modellers.*

## Results

Descriptive statistics in addition to the results of the group specific analyses based on t-tests can be found in Table 1.

*H1*. T-test analysis revealed no significant differences regarding PM quality between inexperienced and experienced modellers.

**Figure 2:**
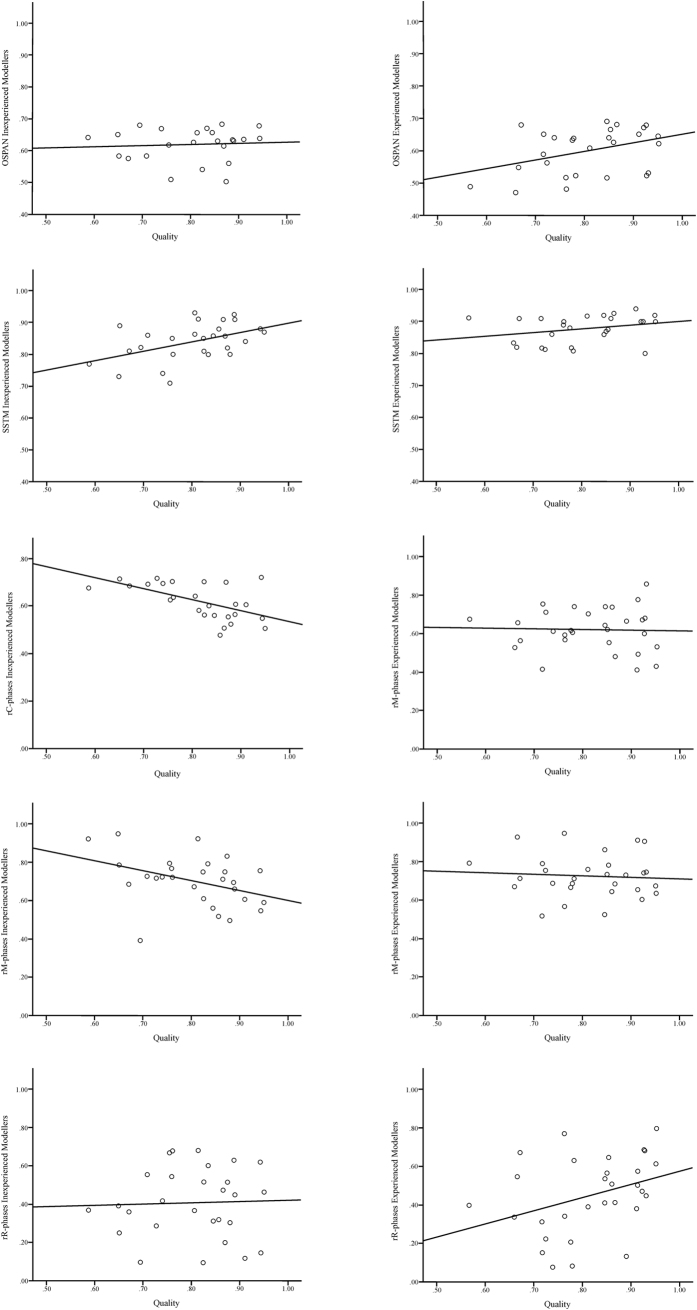
Depicted are the plots of the relations between PM quality (x-axis), OSPAN, SSTM, rC-, rM-, and rR-phases (y-axis) for inexperienced and experienced modellers. OSPAN: operation span; SSTM: spatial short-term memory; rC-phases: ratio of comprehension phases; rM-phases: ratio of modelling phases; rR-phases: ratio of reconciliation phases.

*H2*. T-test analyses showed that inexperienced and experienced modellers did not significantly differ in ability to hold and simultaneously process information, measured with the OSPAN task. However, experienced modellers were significantly better in integrating information elements and their relations, measured with the SSTM task. In a multiple regression analysis, where quality was regressed on the two WM functions we found that only relational integration (SSTM) was significantly related to quality, in both groups (inexperienced modellers: *R^2^* = 0.227, *F*(2,21) = 4.56, *p* = 0.024; *f*^*2*^ = 0.479; OSPAN: *β* = *−*0.027, *p* = 0.889; SSTM: *β* = 0.570, *p* = 0.007; experienced modellers: *R^2^* = 0.488, *F*(2,23) = 10.01, *p* = 0.001; *f*^*2*^ = 0.953; OSPAN*: β* = 0.272, *p* = 0.121; SSTM: *β* = 0.551, *p* = 0.004). The corresponding data distribution of the two WM functions with quality can be found in Fig. 2.

*H3*–*H5*. First, we analysed group differences regarding the total processing time and number of C-, M-, and R-phases before we focussed on group differences regarding rC-, rM-, and rR-phases and their relations to PM quality. T-test analyses revealed that inexperienced modellers needed significantly more time for creating the PM compared to experienced modellers. Regarding the number of C-, M-, and R-phases we found, that inexperienced modellers showed significantly more C- and M-phases compared to experienced modellers. Both modeller groups did not significantly differ regarding the R-phases. Further, no significant differences in rC-, rM-, and rR-phases were found. In a multiple regression analysis, where quality was regressed on the three r-phases we found, that rC-phases were significantly related to PM quality in inexperienced modellers (*R^2^* = 0.451, *F*(3, 25) = 6.03, *p* = 0.004; *f*^*2*^ = 0.821; rC-phases: *β* = *−*0.646, *p* = 0.011; rM-phases: *β* = *−*0.035, *p* = 0.890; rR-phases: *β* = *−*0.112, *p* = 0.574). In experienced modellers rR-phases were significantly related to quality (*R^2^* = 0.326, *F*(3, 27) = 3.88, *p* = 0.022; *f*^*2*^ = 0.484; rC-phases: *β* = 0.244, *p* = 0.248; rM-phases: *β* = *−*0.415, *p* = 0.065; rR-phases: *β* = 0.612, *p* = 0.003). Corresponding data distributions of the three PPM phases with PM quality can be found in Fig. 2.

To summarize, our results revealed that higher abilities in relational integration and a lower number of comprehension phases per time increased PM quality for inexperienced modellers. In experienced modellers, higher abilities in relational integration and a higher number of reconciliation phases per time increase PM quality. Finally, we compared the beta weights of the regression analyses between inexperienced and experienced modellers. We found no significant differences in the beta weights between the two modeller groups (OSPAN: *p*_*βi*-*βe*_ = 0.208; SSTM: *p*_*βi*-*βe*_ = 0.268; rM-phases: *p*_*βi*-*βe*_ = 0.127; rR-phases: *p*_*βi*-*βe*_ = 0.056), except for rC-phases (*p*_*βi*-*βe*_: = 0.010). These results indicate that only the relation between the rC-phases and PM quality was significantly stronger for inexperienced modellers compared to experienced modellers.

## Discussion

The present study investigated the role of different WM functions and PPM phases in process modelling. Our results indicate that relational integration is strongly related to PM quality in inexperienced and experienced modellers. Additionally, lower rC-phases in inexperienced modellers and higher rR-phases in experienced modellers seem to be an indicator for PMs of higher quality.

### H1 – PM quality differences

In our study, inexperienced and experienced modellers created PMs of the same quality, a finding that contradicts H1. There are at least three explanations for this discrepancy. First, we set no time limit for creating the PM. Compared to experienced modellers, inexperienced modellers needed significantly more time for creating the PM. We assume that processing time can support mental processes like cognitive control (e.g. examining whether a specific activity was set or a PM solution fits with the text content), problem solving processes (e.g. mentally creating one or more solution alternatives), and even can reduce stress induced error behaviour (e.g. no time pressure for thinking, modelling, reorganizing, and laying out the PM; see Arnsten^54^). Second, task complexity might have been too low, i.e. the number of notational elements required to create the solution model was rather small. This explanation was supported by the relatively high quality scores of .82 and .80 for experienced and inexperienced modellers, respectively. Third, we investigated expertise in its earliest phase. That is, even though not expected, our intensive training session for the inexperienced modellers might have led to a steep learning progress resulting in similar modelling performance between the two modeller groups (at least under our modelling conditions; but see studies into the power and exponential functions of learning^55^,^56^). In this context it is even more interesting to see that relational integration in both groups and different PPM phases are related to similar PM quality outputs. In other words, differences between the two modeller groups appeared in the PPM phases, whereby relational integration was a common predictor for PM quality in both groups.

### H2 - Working memory and PM quality

The significant relation between relational integration and PM quality is in line with existing studies which show that relational integration represents a central predictor for reasoning ability and fluid intelligence^37^,^43^. High abilities in holding the relations of verbal information and modelling elements (e.g. operators) in an active state seem to reduce modelling errors and increase the probability of representing the model in a semantically and syntactically correct manner^57^. The relevance of relational integration in process modelling seems to be valid for inexperienced and experienced modellers. This finding is interesting, because one could argue that training should reduce the dependency of our cognitive system on WM^51^. This is, among other things, because training/experience leads to accumulation of process modelling knowledge which is organized and stored in chunks in long-term memory. These knowledge chunks are easily accessible by only a few cues. As a result, high amounts of process modelling knowledge are at hand and ready for a relatively automatic application^48^. However, there are at least two reasons for our results. First, our experienced modellers cannot be considered as modelling experts. Regarding this, WM or specific WM functions should play a role, because WM reveals its predictive power especially in new situations, contexts, and tasks^31^,^58^,^59^,^60^. Second, even in experienced modellers WM represents a central predictor for task performance. Existing studies show that training and the application of mental strategies can circumvent existing short-term memory capacity limitations and reduce the predictive power of IQ^61^. However, contrary to these findings a recent meta-analysis indicates that deliberate practice has a significant impact only in specific domains like games, music, and sports, and is less important in contexts like education and professions^62^. For Macnamara *et al*.^62^ WM and IQ represent strong candidates for explaining variance in performance that deliberate practice cannot explain^53^. Our study supports this view and extends it assuming that specific WM functions may be obtained while the predictive power of others is weakened by training depending on the task context (e.g. with and without the possibility of information externalization).

Finally, we did not find a relation between the function of holding and processing and quality, even though this WM function is, like relational integration, strongly related to a host of higher-order cognitive abilities (e.g. problem solving, text comprehension, intelligence^29^,^30^,^31^,^32^). One possible explanation for this finding is that WM capacity is often defined as ability to actively maintain information outside the focus of attention, while simultaneously other information is processed within the focus of attention^33^. In our context, the textual process description, modelling operators, and the created model were visible throughout the whole processing time. Therefore, information inside and outside the focus of attention could be continuously refreshed, possibly making this specific WM function less relevant^26^,^27^.

### H3 to H5 - PPM phases and PM quality

Our results indicate that low amounts of rC-phases, i.e. phases in which no operator applications took place, increased the quality of a PM. We reason that high WM capacity modellers, presumably modellers with higher abilities in relational integration create PMs of higher quality with less rC-phases. Training of the specific cognitive abilities like relational integration and/or pre-existing process modelling knowledge, which helps to circumvent existing WM capacity limitations^51^, can serve as arguments why experienced modellers showed significantly less rC-phases compared to inexperienced modellers.

Regarding reconciliation phases, we proposed that renaming and layout activities increased the understandability and subsequently the quality of the PM. This view is supported by our results. We assume that reconciliation can be understood as an external thinking strategy. After externalization of model information, visual organization of model elements can facilitate the problem solving process through, for instance, validation and/or reinterpretation processes^57^. For example, the reorganization of existing model elements can help to create subsequent model elements more effectively and accurately, reducing superfluous modelling steps and cognitive load. However, it is important to note that we did not classify our participants into various types of modellers. For instance, we did not differentiate between modellers (i) creating correct models from the beginning without the need for renaming and layout activities, (ii) continuously incorporating R-phases in process modelling, and (iii) applying R-phases not until the end. Additionally, even though higher rR-phases led to PMs of higher quality, one cannot exclude that different modelling styles can be equally effective, for instance, modellers creating high quality PMs without the need for a higher number of R-phases. Applying such a modelling behaviour would require a precise depiction of the PM in mind and an accurate translation of the mind’s model into an externalized model.

Our results with respect to rC- and rR-phases can explain why rM-phases did not seem to be related to PM quality in both groups. We assume that PM quality heavily depends on the amount of effort spent on the comprehending the textual process description and the effectiveness of visually and semantically organizing the PM in order to correctly transform the task description into a high quality PM. The simple act of moving an already mentally chosen element from the operator toolbox into the modelling area seemed not to be of relevance in this process. However, it should be noted that these findings do not rule out a possible role of modelling phases in creating PMs of high quality. The impact of the modelling phases on PM quality could be revealed by investigating their relations with specific quality measures. For instance, it is conceivable that there is a relation between modelling phases and syntactic quality^63^.

In this context, the question arises how WM is related to the three PPM phases, highlighting a central role of WM in the creation process of a PM. Based on the outline above one can hypothesize that WM is negatively related to rC-, rM-, and positively related to rR-phases because WM plays a central role in integrating, creating and restructuring information elements. Testing this, we found that experienced modellers with lower relational integration abilities showed more rC-phases (*R^2^* = 0.319, *F*(2, 26) = 5.61, *p* = 0.010; *f*^*2*^ = 0.468; OSPAN*: β* = *−*0.095, *p* = 0.602; SSTM: *β* = *−*0.524, *p* = 0.008). This finding is in line with existing studies which show negative relations between WM and, for instance, reading comprehension and processing time^64^,^65^, problem solving^31^, and encoding of new information^59^. Additionally, we found a significant regression model for the rR-phases in the experienced modellers group (*R^2^* = 0.221, *F*(2, 26) = 3.40, *p* = 0.050; *f*^*2*^ = 0.284; OSPAN*: β* = *−*0.265, *p* = 0.182; SSTM: *β* = *−*0.305, *p* = 0.126). Interpretation of this result is not straightforward. On the one hand, we showed that high rC phases lead to PMs of higher quality. On the other hand, our findings indicate that high WM capacity individuals had less rC-phases. This result confirms the assumption that rR-phases play a role as cognitive strategy to circumvent WM limitations, and therefore decrease cognitive load.

Finally, our findings indicate that inexperienced and experienced modellers differed in their ability to integrate information elements and their relations. Whether this finding is a training and/or population specific effect cannot be solved with our study. It is a matter of ongoing debate whether WM and its functions are trainable and transferable to other cognitive abilities like fluid intelligence and/or problem solving^66^,^67^.

### Limitations

A possible limitation of our study was that we investigated process modelling under laboratory settings. A methodological advantage of this approach is the possibility to control for interacting factors, e.g., task instructions, task complexity, working conditions (e.g. use of external memory aids like sketches), and experience. However, a disadvantage is that laboratory experiments lack ecological validity. This means that in contrast to laboratory settings, modellers in applied settings integrate relevant information from various sources (e.g. a conversation, piece of paper) that is presented in a less well-structured way. Additionally, future research has to confirm our results and delineate them from performance in expert modellers (e.g. based on a minimum number of created PMs). This has to be investigated not only for additional measures of PM quality (e.g. syntactic errors) but also for the PPM. Finally, when interindividual modelling differences are investigated additional factors, beside practice and cognitive abilities, have to be considered (e.g., personality factors such as “passion” or achievement motivation; handling performance anxiety and “choking” under pressure; executive functions such as memory updating and inhibition of irrelevant information; and genetic variances)^53^.

### Implications

Potential implications of our results can be seen in the construction of modelling environments in which the modeller together with an intelligent modelling software agent creates PMs of higher quality, much more effectively. For instance, an agent could detect the individual modelling behaviour and dynamically organize and support specific tool features to optimally structure modelling sessions especially in early PPM phases^68^. At the same time this agent can flexibly shape the modelling environment in a way that it adapts automatically to the cognitive (WM) capacity constraints of a modeller through, for instance, WM unloading opportunities in certain PPM phases (e.g. C-phases). Further, our results can have an impact on teaching the craft of modelling. For instance, students creating PMs can be monitored on the fly, immediately identifying problems during the creation process (i.e. in which phase) in order to provide individual support.

## Methods

### Ethics statement

The present research was conducted with approval by the Institutional Review Board of the University of Innsbruck (Faculty of Psychology and Sport Science). All applied methods were carried out in accordance with the approved guidelines. Participants gave written informed consent.

### Participants

Sixty students from the University of Innsbruck took part in the experiment. Experienced modellers (22 males, 8 females, mean age = 26.76 years, *SD* = 2.90) represented computer science students familiar in process modelling (through process modelling lectures and/or the composition of a bachelor or master thesis on process modelling). Inexperienced modellers (16 males, 14 females, mean age = 22 years, *SD* = 4.58) represented psychology students without process modelling experience, but trained in process modelling notation and program handling for the first time.

### Materials

#### Modelling task

Participants of both groups were required to model a lending process based on a textual process description. Participants of both groups were tested in single experimental settings and worked on the same process modelling task (for the full task text: http://bpm.q-e.at/WorkingMemory). No time limit was set. The modelling task was implemented in Cheetah Experimental Platform (CEP)^69^. For a representative quality score we computed a mean global quality score based on two measures, graph edit distance (GED) and activity correctness (AC).

GED quantifies the transformations required to convert one PM to another PM using a series of elementary transformations^24^,^25^. More specifically, node substitutions, i.e. replacing one node by another, node insertions/deletions and edge insertions/deletions are used^24^. By associating each elementary transformation with a cost function, the cost of transforming one PM to another can be calculated. In our context, we calculated the cost of transforming the PM created by a modeller to a model representing the goal state. For this, we conducted the following steps. First, we manually constructed a mapping of the activities in the PM to the activities of the goal state model. This is necessary because participants differ in the way they label the activities. Second, the GED was calculated using the algorithm described in Dijkman *et al*.^24^ to obtain the elementary transformations for each PM. Third, the graph edit similarity was calculated to obtain a normalized result between 0.0 and 1.0, where 1.0 constitutes an exact match of the two PMs, i.e. the PM and the goal state model (Fig. 1A).

Regarding AC we applied a grading scheme measuring which activities described in the textual process description were correctly identified and represented in the PM. Therefore, we compared the activities depicted in the end model of each participant with the goal state model (Fig. 1A). In order to obtain the overall quality of the PM, each correct activity was scored by one point. The overall AC score of the PM was defined as the sum of the correct modelling interactions. For the purpose of normalizing the AC score and making it comparable with the GED score we divided the AC sum score by the maximum number of correct activities. Therefore, we obtained an AC score between 0.0 and 1.0, where 1.0 indicated that all activities were present. To reduce measure specific variance we calculated a general quality score. The general quality score was defined as the mean of the normalized GED and AC scores.

The number of C-, M-, and R-phases were automatically identified based on an algorithm described in Pinggera *et al*.^22^. For further analysis we calculated a ratio C-, M-, and R score. For this, we divided the number of C-, M-, and R- phases by the individuals’ total processing time (rC-, rM-, and rR-phases).

#### Working memory

We measured the WM function of holding and processing with the OSPAN task, and relation integration with the SSTM task. Both WM tasks were taken from the WM test battery of Lewandowsky, Oberauer, Yang, and Ecker^40^. The tasks ran on MATLAB 7.10.0.499 (R2010a) with the Psytoolbox Version 3^70^,^71^.

#### OSPAN task

Participants saw an alternating sequence of arithmetic equations (e.g. 4 + 3 = 7) and consonants that had to be remembered in the correct order for later serial recall. In each trial the list length (i.e. the number of equations-letter alternations) varied between four to eight (3 trials per list length). There were 15 experimental trials and 3 practice trials. Participants’ operation span (OSPAN) score was computed based on the proportion of items recalled correctly. A detailed description of the task can be found in Lewandowsky *et al*.^42^.

#### SSTM task

Participants saw a 10 × 10 grid in which various numbers of dots were presented one by one. Participants were required not to recall the absolute spatial position of the dots, but their spatial relations. The set size (number of dots per trial) was between two to six dots. The task consisted of 30 experimental trials, 6 at each set size. Experimental trials were preceded by two practice trials. Participants´ spatial short-term memory (SSTM) score was computed according to the distance between the learning dots and the generated dots. If the distance was 0 cells, 2 points were gained. If the distance was 1 cell, 1 point was gained. If the distance was further than 1 cell, 0 points were gained. The total score in the SSTM task represented the sum of all scores on all trials divided by the total number of possible points. A detailed description of the task can be found in Lewandowsky *et al*.^42^.

### Procedure

#### Inexperienced modellers

Participants were first informed that the experiment consists of two sessions, (i) a process modelling training and (ii) a testing session. In the first part of the training session participants were prompted to conduct two working memory tasks, OSPAN and SSTM. WM tasks were conducted one by one always after a short task specific introduction through the examiner. After a break of 15 minutes participants were trained in CEP^69^ (see Fig. 1C for the CEP modelling environment). The training consisted of three parts. First, participants got an example-based introduction to process modelling (what is understood by process modelling, “XOR” and “AND” gateways etc.). Second, participants were induced to the CEP environment (the examiner guided through CEP with a simple example PM participants had to create, including layout guidelines and examples for PMs of low quality). Third, participants were required to model a process modelling task themselves. An ideal PM solution for this task was presented at the end of the training session and all open questions were answered. Training took about 90 minutes in a computer laboratory with 25 laptops. Training group size was between 5 and 15 participants. After all participants had finished CEP training, they were prompted to indicate one possible time point for the process modelling testing session. All participants were tested within seven days after training. The process modelling testing session consisted of two parts, a familiarization and a testing phase. Preliminary to the testing phase participants of the inexperienced modellers group were given the possibility to freely use CEP for several minutes to get familiar with the program before they called the examiner that they are now ready for modelling the main experimental task. The examiner started CEP and the participant began working on the modelling task.

#### Experienced modellers

Participants were directly invited to the single testing sessions. Testing sessions were fully identical to the inexperienced modellers group, except that experienced modellers were first tested in the modelling session before they conducted the two WM tasks (OSPAN and SSTM) after a break of 15 min.

### Statistical analyses

For statistical analysis the alpha level was set at .05, testing two-tailed. We first analysed group differences regarding PM quality, the different WM functions, and PPM phases based on t-tests. This was followed by multiple regression analyses where we regressed quality on the two WM functions, and quality on the three PPM phases. Variations in the degrees of freedom were based on missing values and/or due to software malfunction. Outlier analyses were based on boxplot diagrams, calculation of the leverage values and studentized deleted values^72^,^73^.

## Additional Information

**How to cite this article**: Martini, M. *et al*. The impact of working memory and the “process of process modelling” on model quality: Investigating experienced versus inexperienced modellers. *Sci. Rep.* **6**, 25561; doi: 10.1038/srep25561 (2016).

## Figures and Tables

**Table 1:** Descriptive and inference statistics of PM quality, total processing time, WM functions, and PPM phases for inexperienced and experienced modellers.

		Quality	Time	OSPAN	SSTM	C-phases	M-phases	R-phases	rC-phases	rM-phases	rR-phases
Inexperienced	*M*	0.80	29.66	0.61	0.84	18.21	21.04	12.04	0.62	0.70	0.41
	*SD*	0.10	9.73	0.06	0.06	5.74	5.11	6.62	0.08	0.13	0.18
Experienced	*M*	0.82	23.61	0.60	0.88	14.72	16.00	10.48	0.62	0.73	0.45
	*SD*	0.10	8.13	0.07	0.04	5.85	4.76	5.74	0.11	0.11	0.20
Group differences	*t*	−0.47	−2.55	0.84	−2.82	2.27	3.78	0.95	0.05	−0.73	−0.89
	*df*	57	55	50	53	55	53	55	55	56	57
	*p*	0.637	**0.014**	0.407	**0.007**	**0.027**	**0.001**	0.348	0.958	0.471	0.379
	*η^2^*	0.004	0.106	0.014	0.131	0.084	0.212	0.016	0.001	0.001	0.014

Note. M: mean; SD: standard deviation; Time: total processing time in minutes; OSPAN: operation span; SSTM: spatial short-term memory; C-phases: number of comprehension phases; M-phases: number of modelling phases; R-phases: number of reconciliation phases; rC-phases: ratio of comprehension phases; rM: ratio of modelling phases; rR: ratio of reconciliation phases. rC-, rM-, and rR-phases scores are based on the C-, M-, and R-phases divided by individuals´ total processing time in minutes. Bold: significant results, *p* < 0.05.

## References

[b1] Dumas M., van der Aalst W. M. P. & ter Hofstede A. H. M. Process aware information systems: Bridging people and software through process technology. 1–432 (Wiley–Interscience, Hoboken, NJ, 2005).

[b2] Erl T. Service–oriented architecture: Concepts, technology, and design. Ch. 16, 565–586 (Prentice Hall, 2005).

[b3] Montali M. . Declarative specification and verification of service choreographies. ACM Transactions on the Web 4, 1–62 (2010).

[b4] Reijers H. A. & Mendling J. A study into the factors that influence the understandability of business process models. IEEE Trans. Syst. Man Cybern. Part A Syst. Hum. 41, 449–462 (2011).

[b5] Mendling J., Strembeck M. & Recker J. Factors of process model comprehension—findings from a series of experiments. Decis. Support Syst. 53, 195–206 (2012).

[b6] Recker J., Reijers H. A. & Van de Wouw S. G. Process model comprehension: The effects of cognitive abilities, learning style, and strategy. Communications of the Association for Information Systems 34, 199–222 (2014).

[b7] Figl K., Mendling J. & Strembeck M. The influence of notational deficiencies on process model comprehension. J. Assoc. Inf. Syst. 14, 312–338 (2013).

[b8] Mendling J., Reijers H. A. & Recker J. Activity labeling in process modeling: Empirical insights and recommendations. Inf. Syst. 35, 467–482 (2010).

[b9] Recker J., Safrudin N. & Rosemann M. How novices design business processes. Inf. Syst. 37, 557–573 (2012).

[b10] Claes J. . Tying process model quality to the modeling process: The impact of structuring, movement, and speed. Proc. BPM 2012 7481, 33–48 (2012).

[b11] Mendling J. Metrics for process models, Vol. 6, 103–133 (Springer, Berlin, 2008).

[b12] Pinggera J. . How the structuring of domain knowledge helps casual process modelers in *Conceptual modeling* (eds Parsons J., Saeki M., Shoval P., Woo C. & Wand Y.) 445–451 (Springer, Berlin, 2010).

[b13] Newell A. & Simon H. Human problem solving. 53–791 (Prentice Hall, Englewood Cliffs, NJ, 1972).

[b14] Sachse P. . Das Arbeitsgedächtnis als “ Nadelöhr” des Denkens [Working memory, the “bottleneck” of thinking] in Psychologie menschlichen Handels: Wissen und Denken – Wollen und Tun [The psychology of human action: Knowing and thinking – willing and doing] (eds Sachse P. & Ulich E.) 339–367 (Pabst, Lengerich, 2014).

[b15] Wilmont I., Hengeveld S., Barendsen E. & Hoppenbrouwers S. Cognitive mechanisms of conceptual modelling in *Conceptual modeling* (eds Ng W., Storey V. C. & Trujillo C.) 74–87 (Springer, Berlin, 2013).

[b16] Neurauter M. . The influence of cognitive abilities and cognitive load on business process models and their creation. Proc. NeuroIS 2015 10, 107–115 (2015).

[b17] Hoppenbrouwers S. J. B. A., Proper E. H. & van der Weide T. P. A fundamental view on the process of conceptual modeling. Proc. ER 2005 3716, 128–143 (2005).

[b18] Hoppenbrouwers S. J. B. A., Proper E. H. & van der Weide T. P. Formal modelling as a grounded conversation in Proceedings of the 10^th^ international working conference on the language action perspective on communication modelling (eds Goldkuhl G., Lind M. & Haraldson S.) 139–155 (2005).

[b19] Pinggera J. The process of process modeling [doctoral dissertation] (University of Innsbruck, Innsbruck, 2014).

[b20] Reichert M. & Weber B. Enabling flexibility in process-aware information systems. Challenges, Methods, Technologies. 9–489 (Springer, Berlin, Heidelberg, 2012).

[b21] Weske M. Business process management. Concepts, languages, architectures. 3–389(Springer, Berlin, Heidelberg, 2007).

[b22] Pinggera J. . Tracing the process of process modeling with modeling phase diagrams. Proc. ER-BPM 2011 99, 370–382 (2012).

[b23] Claes J., Gailly F. & Poels G. Cognitive aspects of structured process modelling. Proc. CAiSE 2013, Advanced information systems engineering workshops 148, 168–173 (2013).

[b24] Dijkman R., Dumas M. & García-Bauñelos L. Graph matching algorithms for business process model similarity search. Proc. BPM 2009 5701, 48–63 (2009).

[b25] Dijkman R., Dumas M., Van Dongen B., Käärik R. & Mendling J. Similarity of business process models: Metrics and evaluation. Information Systems 36, 498–516 (2011).

[b26] Oberauer K. & Hein L. Attention to information in working memory. Curr Dir. Psychol. Sci. 21, 164–169 (2012).

[b27] Cowan N. . On the capacity of attention: Its estimation and its role in working memory and cognitive aptitudes. Cognitive Psychol. 51, 42–100 (2005). 10.1016/j.cogpsych.2004.12.001 PMC2673732 16039935

[b28] Martini M., Furtner M. R., Maran T. & Sachse P. Information maintenance in working memory: An integrated presentation of cognitive and neural concepts. Front. Syst. Neurosci. 9, 104, doi: 10.3389/fnsys.2015.00104 (2015). 26236205 PMC4500897

[b29] Just M. A. & Carpenter P. A. A capacity theory of comprehension: Individual differences in working memory. Psychol. Rev. 99, 122–149 (1992). 1546114 10.1037/0033-295x.99.1.122

[b30] Kyllonen P. C. & Stephens D. L. Cognitive abilities as determinants of success in acquiring logic skill. Learn. Individ. Differ. 2, 129–160 (1990).

[b31] Conway A. R. A., Cowan N., Bunting M. F., Therriault D. J. & Minkoff S. R. B. A latent variable analysis of working memory capacity, short-term memory capacity, processing speed, and general fluid intelligence. Intelligence 30, 163–184 (2002).

[b32] Hambrick D. Z. & Engle R. W. Effects of domain knowledge, working memory capacity, and age on cognitive performance: An investigation of the knowledge-is-power hypothesis. Cognitive Psychol. 44, 339–387 (2002). 10.1006/cogp.2001.0769 12018938

[b33] Engle R. W. Working memory capacity as executive attention. Curr. Dir. Psychol. Sci. 11, 19–23 (2002).

[b34] Kane M. J., Conway A. R. A., Hambrick D. Z. & Engle R. W. Variation in working memory capacity as variation in executive attention and control in *Variation in working memory* (eds Conway A. R. A., Jarrold C., Kane M. J., Miyake A. & Towse J. N.) 21–48 (Oxford University Press, New York, 2007).

[b35] Unsworth N. & Engle R. W. The nature of individual differences in working memory capacity: Active maintenance in primary memory and controlled search from secondary memory. Psychol. Rev. 114, 104–132 (2007). 17227183 10.1037/0033-295X.114.1.104

[b36] Oberauer K., Süß H.-M., Wilhelm O. & Sander R. Individual differences in working memory capacity and reasoning ability in *Variation in working memory* (eds Conway A. R. A., Jarrold C., Kane M. J., Miyake A. & Towse J. N.) 21–48 (Oxford University Press, New York, 2007).

[b37] Oberauer K., Süß H. M., Wilhelm O. & Wittmann W. W. Which working memory functions predict intelligence. Intelligence 36, 641–652 (2008).

[b38] Wilhelm O., Hildebrandt A. & Oberauer K. What is working memory capacity, and how can we measure it? Front. Psychol. 4, 433, doi: 10.3389/fpsyg.2013.00433 (2013). 23898309 PMC3721021

[b39] Turner M. & Engle R. W. Is working memory capacity task dependent? J. Mem. Lang. 28, 127–154 (1989).

[b40] Unsworth N., Heitz R. P., Schrock J. C. & Engle R. W. An automated version of the operation span task. Behav. Res. Methods 37, 498–505 (2005). 16405146 10.3758/bf03192720

[b41] Oberauer K. Die Koordination kognitiver Operationen: Eine Studie über die Beziehung zwischen Intelligenz und “working memory” (The coordination of cognitive operations: A study on the relation of intelligence and “working memory”. Zeitschrift für Psychologie 201, 57–84 (1993).

[b42] Lewandowsky S., Oberauer K., Yang L. & Ecker U. A working memory test battery for MATLAB. Behav. Res. Methods 42, 571–585 (2010). 20479189 10.3758/BRM.42.2.571

[b43] Oberauer K., Süß H. M., Wilhelm O. & Wittmann W. W. The multiple faces of working memory: Storage, processing, supervision, and coordination. Intelligence 31, 167–193 (2003).

[b44] Friedman N. P. . Not all executive functions are related to intelligence. Psychol. Sci. 17, 172–179 (2006). 16466426 10.1111/j.1467-9280.2006.01681.x

[b45] Zugal S., Pinggera J. & Weber B. Assessing process models with cognitive psychology. Proc. EMISA 2011 P-190, 177–182 (2011).

[b46] Wilmont I., Hengeveld S., Barendsen E. & Hopenbrouwers S. Cognitive mechanisms of conceptual modelling - how do people do it? Conceptual Modelling, Lecture Notes in Computer Science 8217, 74–87 (2013).

[b47] Pinggera J. . The modelling mind: Behavior patterns in process modelling. Proc. BPMDS 2014 175, 1–16 (2014).

[b48] Recker J., Reijers H. & van de Wouw S. Process model comprehension: The effects of cognitive abilities, learning style, and strategy. Communications of the association for information systems 34, 199–222 (2014).

[b49] Ericsson K. A., Charness N., Feltovich P. J. & Hoffman R. R. The Cambridge handbook of expertise and expert performance. 3–789 (Cambridge University Press, Cambridge, 2006).

[b50] Holyoak K. J. Symbolic Connectionism: Toward third-generation theories of expertise in Toward a general theory of expertise: Prospects and limits (eds Ericsson K. A. & Smith J.) 301–335 (Cambridge University Press, Cambridge, 1991).

[b51] Ericsson K. A. & Kintsch W. Long-term working memory. Psychol. Rev. 102, 211–245 (1995). 7740089 10.1037/0033-295x.102.2.211

[b52] Fincher-Kiefer R., Post T. A., Greene T. R. & Voss J. F. On the role of prior knowledge and task demands in the processing of text. J. Mem. Lang. 27, 416–428 (1988).

[b53] Hambrick D. Y., Macnamara B. N., Campitelli G., Ullen F. & Mosing M. A. Beyond born versus made: A new look at expertise. Psychol. Learn. Motiv. 64, 1–55 (2016).

[b54] Arnsten A. F. T. Stress signalling pathways that impair prefrontal cortex structure and function. Nat. Rev. Neurosci. 10, 410–422 (2009). 19455173 10.1038/nrn2648 PMC2907136

[b55] Newell A. & Rosenbloom P. S. Mechanisms of skill acquisition and the law of practice in *Cognitive skills and their acquisition* (ed. Anderson J. R.) 1–55 (Erlbaum, Hillsdale, NJ, 1981).

[b56] Heathcote A., Brown S. & Mewhort D. J. K. The power law repealed: The case for an exponential law of practice. Psychon. Bull. Rev. 7, 185–207 (2000). 10.3758/bf03212979 10909131

[b57] Sachse P., Hacker W. & Leinert S. External thought – Does sketching assist problem analysis? Appl. Cognit. Psychol. 18, 415–425 (2004).

[b58] Rosen V. M. & Engle R. W. Forward and backward serial recall. Intelligence 25, 37–47 (1997).

[b59] Rosen V. M. & Engle R. W. The role of working memory capacity in retrieval. J. Exp. Psychol. Gen. 126, 211–227 (1997). 9281831 10.1037//0096-3445.126.3.211

[b60] Rosen V. M. & Engle R. W. Working memory capacity and suppression. J. Mem. Lang. 39, 418–436 (1998).

[b61] Bilalic M., McLeod P. & Gobet F. Does chess need intelligence? – A study with young chess players. Intelligence 35, 457–470 (2007).

[b62] Macnamara B. N., Hambrick D. Z. & Oswald F. L. Deliberate practice and performance in music, games, sports, education, and professions: A meta-analysis. Psychol. Sci. 25, 1608–1618 (2014). 24986855 10.1177/0956797614535810

[b63] Soffer P., Kaner M. & Wand Y. Towards understanding the process of process modeling: Theoretical and empirical considerations. Business Process Management Workshops. Lecture Notes in Business Information Processing 99, 357–369 (2012).

[b64] Budd D., Whitney P. & Turley K. J. Individual differences in working memory strategies for reading expository text. Mem. Cognition 23, 735–748 (1995). 10.3758/bf03200926 8538446

[b65] Daneman M. & Carpenter P. A. Individual differences in working memory and reading. J. Verb. Learn. Verb. Beh. 19, 450–466 (1980).

[b66] Shipstead Z., Redick T. S. & Engle R. W. Is working memory training effective? Psychol. Bulletin 138, 628–654 (2012). 10.1037/a0027473 22409508

[b67] Jaeggi S. M. & Buschkuehl M. Training working memory in Working memory. The connected intelligence (eds T. P. Alloway & R. G. Alloway) 277–285 (Taylor & Francis, Hove, East Sussex, 2013).

[b68] Recker J. “Modeling with tools is easier, believe me” – The effects of tool functionality on modeling grammar usage beliefs. Inf. Syst. 37, 213–226 (2012).

[b69] Pinggera J., Zugal S. & Weber B. Investigating the process of process modeling with Cheetah Experimental Platform. Proc. ER-POIS 2010 10, 13–18 (2010).

[b70] Brainard D. H. The psychophysics toolbox. Spat. Vis. 10, 433–436 (1997). 9176952

[b71] Pelli D. G. The Video Toolbox software for visual psychophysics: Transforming numbers into movies. Spat. Vis. 10, 437–442 (1997). 9176953

[b72] Raymondo J. C. Statistical analysis in the behavioural sciences. 3–335 (Boston, McGraw-Hill College, 1999).

[b73] Tabachnick B. G. & Fidell L. S. Using multivariate statistics 5th edn, 3–390 (Boston, Pearson Education, 2007).

