# Corrigendum: The impact of working memory and the “process of process modelling” on model quality: Investigating experienced versus inexperienced modellers

**DOI:** 10.1038/srep28721

**Published:** 2016-07-20

**Authors:** Markus Martini, Jakob Pinggera, Manuel Neurauter, Pierre Sachse, Marco R. Furtner, Barbara Weber

Scientific Reports 6: Article number: 2556110.1038/srep25561; published online: 05092016; updated: 07202016

In this Article, Jakob Pinggera, Manuel Neurauter and Barbara Weber are incorrectly affiliated with ‘Department of Psychology, University of Innsbruck, Innrain 52, 6020 Innsbruck, Austria.’ The correct affiliations are listed below:

Jakob Pinggera and Manuel Neurauter

Department of Computer Science, Technikerstraße 21a, 6020 Innsbruck, Austria.

Barbara Weber

Department of Applied Mathematics and Computer Science, Technical University of Denmark, Anker Engelunds Vej 1, 2800 Kgs. Lyngby, Denmark.

Department of Computer Science, Technikerstraße 21a, 6020 Innsbruck, Austria.

